# ADAPTING EDUCATION ON ELDERSPEAK COMMUNICATION FOR FORMAL CAREGIVERS IN THE HOSPITAL SETTING

**DOI:** 10.1093/geroni/igae098.2911

**Published:** 2024-12-31

**Authors:** Clarissa Shaw, Katie Buchheit

**Affiliations:** University of Iowa, Iowa City, Iowa, United States; University of Iowa, Iowa City, Iowa, United States

## Abstract

Elderspeak is communication to older adults that sounds like babytalk and perpetuates rejection of care. The Changing Talk (CHAT) intervention educates nursing home staff on elderspeak communication and effectively reduces both elderspeak by nursing home staff and rejection of care by residents with dementia. The purpose of this study was to adapt CHAT to CHAT-Acute and pilot-test the new educational program in the hospital setting. To create CHAT-Acute, we piloted CHAT with hospital nursing staff. Qualitative feedback was largely positive indicating that elderspeak was a new and important concept, virtual discussions were helpful, and elderspeak identification activities were valuable. Suggested changes included more challenging content, greater focus on the impact of elderspeak on behavior, and condensed modules with less repetition. Within the modules nearly all participants skipped activities with only one participant completing all in-module activities. We adapted CHAT to CHAT-Acute by adding hospital specific content, adjusting the language to reflect acute care, filming new exemplar videos of care encounters, updating the overall format and activities, and shortening the content for increased acceptability. CHAT-Acute was then piloted with N=57 hospital nursing staff. Confidence in dementia care measured by the CODE scale increased significantly from before to after the modules (p=.002). Greater than 90% of participants agreed that they would use the training in their job and were satisfied overall. Comments from nursing staff included: “eye opening discussion on how we speak to patients” and “it was beneficial because if gave examples and scenarios that can be applied to my work.”

